# Activation of the basal cell carcinoma pathway in a patient with CNS HGNET-BCOR diagnosis: consequences for personalized targeted therapy

**DOI:** 10.18632/oncotarget.13092

**Published:** 2016-11-04

**Authors:** Claudia Paret, Johanna Theruvath, Alexandra Russo, Bettina Kron, Khalifa El Malki, Nadine Lehmann, Arthur Wingerter, Marie A. Neu, Aslihan Gerhold-Ay, Wolfgang Wagner, Clemens Sommer, Torsten Pietsch, Larissa Seidmann, Jörg Faber

**Affiliations:** ^1^ Section of Pediatric Oncology, Children's Hospital, University Medical Center of The Johannes Gutenberg University Mainz, Germany; ^2^ Institute of Medical Biostatistics, Epidemiology and Informatics (IMBEI), University Medical Center of The Johannes Gutenberg University Mainz, Germany; ^3^ Section of Pediatric Neurosurgery, Department of Neurosurgery, University Medical Center of The Johannes Gutenberg University Mainz, Germany; ^4^ Institute of Neuropathology, University Medical Center of The Johannes Gutenberg University Mainz, Germany; ^5^ Department of Neuropathology, University of Bonn, Germany; ^6^ Institute of Pathology, University Medical Center of The Johannes Gutenberg University Mainz, Germany

**Keywords:** HGNET-BCOR, BCOR, WNT, GLI, AXIN

## Abstract

High grade neuroepithelial tumor of the central nervous system with *BCOR* alteration (CNS HGNET-BCOR) is a recently described new tumor entity with a dismal prognosis. The objective of this study was to identify and validate pathways deregulated in CNS HGNET-BCOR as basis for targeted therapy approaches.

We characterized the *BCOR* alteration in a pediatric patient with CNS HGNET-BCOR diagnosis by Sanger sequencing and demonstrated an elevated *BCOR* expression by qRT-PCR and western blot. By whole transcriptome sequencing and Ingenuity Pathway Analysis, we identified the activation of the Sonic Hedgehog (SHH) and of the WNT signaling pathway in two different regions of the primary tumor and of one inoculation metastasis compared to normal brain. We validated the activation of the SHH and of the WNT pathway by qRT-PCR analysis of *GLI1* and *AXIN2* respectively. *GLI1* and *AXIN2* were upregulated in the primary tumor and in two inoculation metastases compared to normal brain. Mutational analysis of *SMO*, *PTCH1* and *SUFU*, three key components of the SHH pathway, revealed a Single Nucleotide Polymorphism (SNP) in *PTCH1* (rs357564). We tested the effect of the GLI-inhibitor arsenic trioxide (ATO) on a short-term cell culture isolated from the metastasis. ATO was able to reduce the viability of the cells with an IC_50_ of 1.3 μM.

In summary, these results provide functional evidence of altered *BCOR* expression and homogeneous coactivation of both the SHH and WNT signaling pathways, building the basis for potential novel therapeutic approaches for patients with a CNS HGNET-BCOR diagnosis.

## INTRODUCTION

Recently, four new molecular CNS tumor entities, which were formerly diagnosed with diverse histological diagnoses, have been described [1]. One of them is CNS high grade neuroepithelial tumor with BCOR alteration (CNS HGNET-BCOR). These tumors predominantly affect children and are characterized by somatic internal tandem duplications (ITDs) in the C-terminus of the BCL-6 co-repressor (BCOR) gene and by *BCOR* overexpression [1]. Preliminary survival data suggest that the CNS HGNET-BCOR entity has poor overall survival [1]. The same duplication in *BCOR* has recently been described in clear cell sarcoma of the kidney (CCSK) [2, 3].

BCOR was originally identified in 2000 as an interacting corepressor of BCL6 [4]. BCOR interacts with class I and II histone deacetylases (HDACs) and it is associated with a large transcriptional regulatory complex that includes Polycomb proteins inducing a repressive chromatin state [4–6]. While germline *BCOR* mutations are responsible for the X-linked oculo-facio-cardio-dental (OFCD) syndrome, somatic alterations have been reported in different human cancers including retinoblastoma, medulloblastoma and leukemia [7–10]. Somatic mutations tend to accumulate on the C-terminal side of the protein, underlying the importance of this region for BCOR function [11].

Sturm et al. identified several deregulated pathways specific for CNS HGNET-BCOR [1]. Among them, the Sonic Hedgehog (SHH) and the WNT signaling pathways were reported to be activated. The WNT and the SHH pathways interact with each other in various cell types and organs eliciting opposing or synergistic cellular effects [12, 13]. Particularly, in basal cell carcinoma, the canonical WNT/beta-catenin signaling is required for SHH pathway-driven tumorigenesis [14].

Several drugs blocking the SHH and the WNT pathways are currently being tested in clinical trials and they could become relevant targeted therapies for CNS HGNET-BCOR. The work of Sturm et al. [1] was based on the microarray data and no further validation of the activated pathways was performed. In order to facilitate the selection of molecular targets, we performed a comprehensive molecular characterization of the primary tumor and the inoculation metastases of a pediatric patient with CNS HGNET BCOR diagnosis and isolated a primary cell culture from its metastasis. In this work we showed elevated BCOR expression at the protein level in CNS HGNET-BCOR for the first time. We described and validated the upregulation of several components and the molecular targets of the SHH and WNT pathway and provided first evidences for the relevance of arsenic trioxide (ATO) in the treatment of these patients.

## RESULTS

### Clinical description

**Figure 1:**
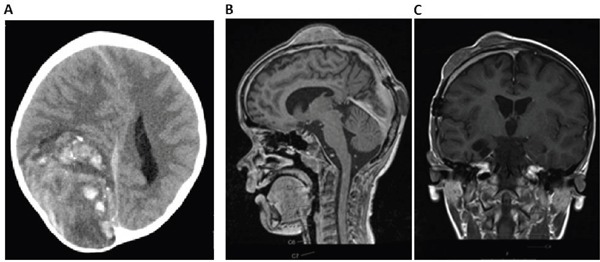
Imaging of CNS HGNET-BCOR primary tumor and metastasis **A.** cCT scan of the primary tumor reveals a 92 x 61 x 87 mm large tumor in the right parieto-occipital lobe. **B-C.** cMRI shows three inoculation metastases on the skullcap.

A 6 year old, male patient was transferred to our hospital due to a large (92 x 61 x 87 mm) hemorrhagic tumor in the right parieto-occipital lobe (Figure 1A). The tumor was macroscopically completely resected and the first local histopathological report was suggestive of a high grade malignant glioma (anaplastic astroblastoma with the differential diagnosis of glioblastoma). The reference pathology laboratory was also unable to come to a definite diagnosis and referred to it as a malignant, partly neuroepithelial tumor. The postoperative staging scans revealed no metastases. With a presumed diagnosis of a malignant glioma, we initiated treatment according to the HIT HGG protocol (cranial irradiation with 59.4 Gy in 30 fractions with concomitant oral temozolamide chemotherapy). Meanwhile, his FFPE tumor sample was analyzed by the Molecular Neuropathology 2.0 diagnostic pipeline and the 450k methylation array analysis revealed a “primitive neuroectodermal tumor with WNT-like subtype”. Due to these novel findings, we added 4 cycles of chemotherapy with vincristine, cisplatin and CCNU according to the HIT-Med protocol. After 4 cycles of chemotherapy the boy developed three inoculation metastases at his skullcap (Figure 1B-1C). Resection of the metastases was performed and the analysis of these samples revealed the same tumor entity. The patient is currently receiving radiotherapy of the three metastatic lesions as relapse therapy

### Histopathology of CNS HGNET-BCOR

**Figure 2:**
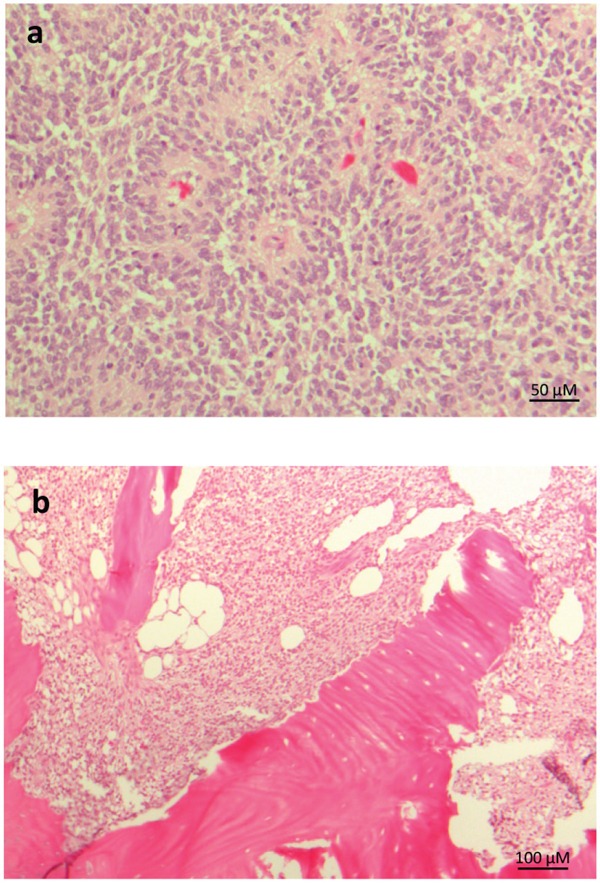
Representative histopathology of CNS HGNT-BCOR HE staining of the primary tumor **a.** and a metastasis **b.** Pseudorosettes are lost in the metastasis.

The histopathology features of CNS HGNET-BCOR were already described [1]. The tumor showed perivascular anuclear zones, which sometimes resemble astroblastic or ependymal architectures (Figure 2a). The cellular morphology of a metastasis was similar to the primary tumor, whereas the perivascular pseudorosettes were lost (Figure 2b).

### Detection of somatic ITDs in primary tumor and metastasis of CNS HGNET-BCOR

**Figure 3:**
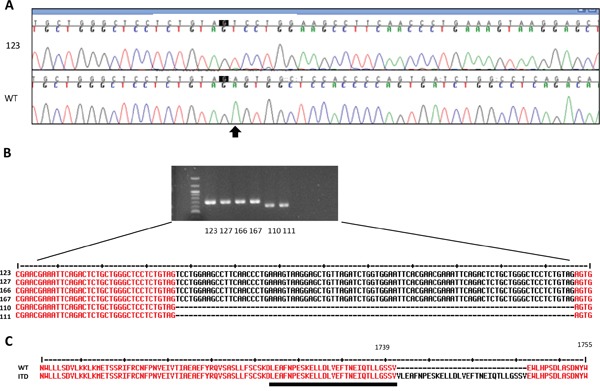
Detection of somatic ITDs in *BCOR* **A.** The sequence of exon 15 of *BCOR* was analyzed by Sanger sequencing in the genomic DNA from blood (WT) or from the primary tumor (123). The arrow indicates the start of the ITD. **B.** RT-PCR showing an aberrant *BCOR* product in 2 regions of the primary tumor (123,127) and two metastases (166, 167) compared to normal brain (110 and 111). The ITD was confirmed by Sanger sequencing. **C.** Protein sequence of the ITD and of normal BCOR (WT). The WT region duplicated in the ITD is underlined. The amminoacid positions according to the sequence Q6W2J9-1 (UniProtKb) are indicated.

Due to the novel findings in the 450k methylation array analysis, we confirmed the diagnosis by sequencing the *BCOR* gene (Figure 3A). No germline mutation could be detected. In contrast, we detected a large insertion in the exon 15 of the primary tumor sample. Only the aberrant product was detected in the tumor sample, which can be explained by the fact that *BCOR* is located on the X chromosome and therefore male individuals carry only one allele. We also confirmed the presence of the ITD at the cDNA level (Figure 3B). The wild-type allele was virtually undetectable in four tumor samples of this patient, suggesting that the *BCOR* ITD is homogenously present in the tumor cells. This can indicate either an early event, or a growth advantage for the tumor cells harboring the *BCOR* ITD. The ITD in *BCOR* was identical with the internal tandem duplication in *BCOR* recently described by Sturm et al. [1] and in clear cell sarcoma of the kidney [2, 3] and consisted in the insertion of one nucleotide and the duplication of 89 nucleotides, leading to an elongated protein (Figure 3C).

### BCOR is overexpressed in CNS HGNET-BCOR

**Figure 4:**
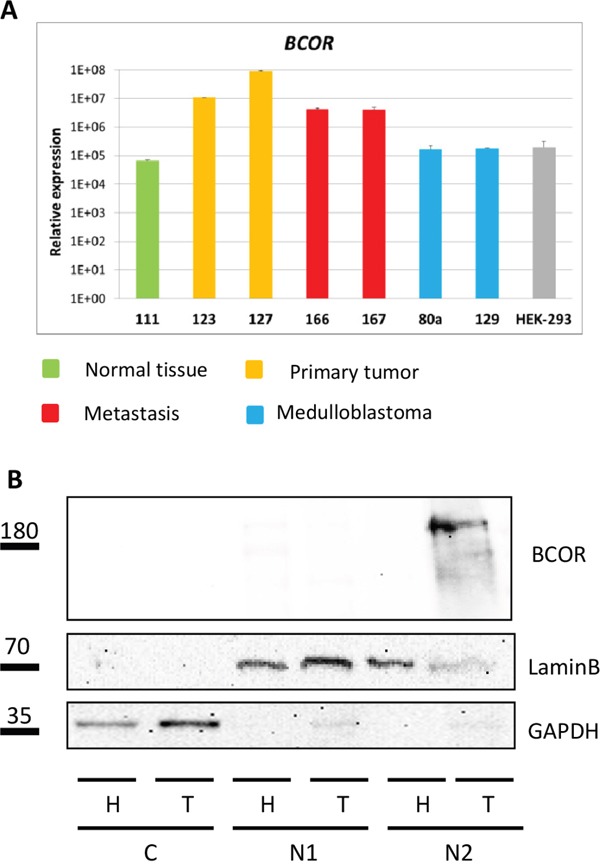
*BCOR* is overexpressed in CNS HGNET-BCOR **A.** *BCOR* expression was analyzed by qRT-PCR in 2 regions of the primary tumor (123,127, in yellow), two metastases (166, 167, in red), normal parietal brain (111, in green), two medulloblastoma (80A, 129, in blue) and HEK-293 cells (grey). After normalization to the housekeeping gene *HPRT1*, the relative quantification value was expressed as 2^−ΔΔCt^. Expression analysis was done in triplicates. Standard deviation is indicated. **B.** Nuclear fractions containing soluble proteins (N1) or insoluble proteins (N2) and cytoplasm (C) isolated from the HEK-293 cell line (H) and the primary tumor (T, no 127) were analyzed by western blot with a BCOR specific antibody or antibodies against different cellular compartments (Lamin B for nuclei, GAPDH for cytoplasm).

The mRNA expression of *BCOR* was found at high levels in CCSK and CNS HGNET-BCOR harboring the *BCOR* ITD [1, 2]. Upregulation of *BCOR* mRNA in ITD-positive samples compared to normal brain, medulloblastoma and HEK-293 cells was confirmed by qRT-PCR (Figure 4A). The *BCOR* mRNA expression was high in both primary tumor samples, suggesting little heterogeneity of the *BCOR* expression. Moreover, a high level of *BCOR* expression was maintained in two inoculation metastases suggesting the *BCOR* expression is not lost during the selection mechanisms required for the adaptation of the tumor cells to a new environment. To assess whether the high transcript levels of *BCOR* are translated into a robust expression of the protein, a western blot analysis was performed (Figure 4B). A BCOR band of the expected size of about 190 KDa was detected only in the non-soluble nuclear fraction of the tumor lysate but not of the HEK-293 cells, which showed lower levels of *BCOR* transcripts (Figure 4A).

### Pathways upregulated in CNS HGNET-BCOR

**Figure 5:**
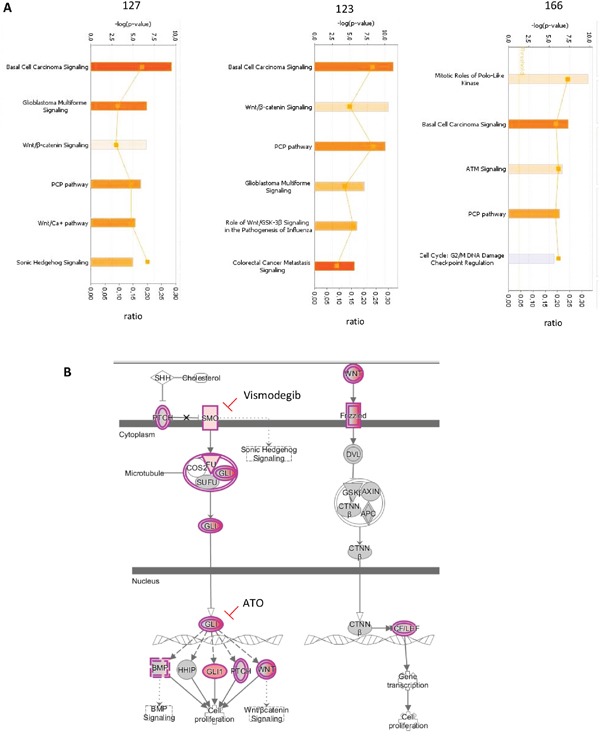
Pathways upregulated in CNS HGNET-BCOR **A.** IPA analysis was performed for two regions of the primary tumor (123 and 127) and of one metastasis (166). The significant Canonical Pathways are displayed along the y-axis. The x-axis displays the −log of p-value which is calculated by Fisher's exact test right-tailed. Only pathways with a-log of p-value of 4.9 are shown. Orange bars predict an overall increase in the activity of the pathway while blue bars indicate a prediction of an overall decrease in activity (z-score). Only absolute z-score more than 0 are shown. The orange points connected by a thin line represent the ratio calculated as follows:# of genes in a given pathway that meet the cutoff criteria, divided by the total # of genes that make up that pathway and that are in the reference gene set. **B.** Representation of the BCC pathway according to IPA. Transcripts upregulated in the primary tumor are in violet. Clinical relevant inhibitors are indicated. The binding of SHH ligand to PTCH1 receptor relieves SMO inhibition and allows the release of the GLI family of transcription factors from a protein complex composed of SUFU, COS2 and FU. GLI proteins accumulate in the nucleus and control the transcription of the SHH target genes BMP, HHIP, GLI1, PTCH and WNT proteins. WNT proteins bind to the Frizzled receptor family and a signal is transduced to the cytoplasmic phosphoprotein DVL. Regulation of beta-catenin (CTNNβ) stability is mediated via a complex of proteins including AXIN, GSK3 and APC. In the nucleus beta-catenin binds and activates members of the T-cell factor (TCF)/lymphoid enhancer binding factor 1 (LEF) transcription factor family inducing the expression of target genes involved in the regulation of proliferation. Abbreviations: SHH = Sonic Hedghog protein, PTCH = patched, GLI = glioma-associated oncogene family zinc finger 1, SMO = Smoothened, SUFU = Suppressor of fused homolog, ATO= arsenic trioxide, COS2 = kinesin-like protein Costal-2, BMP = Bone morphogenetic proteins, HHIP = hedgehog-interacting protein, WNT = Wingless-type MMTV integration site family member, DVL= dishevelled segment polarity protein 1, CTNNβ = beta-catenin, AXIN = axis inhibition protein, APC = Adenomatous polyposis coli, FU= serine/threonine kinase 36, GSK3= glycogen synthase kinase 3.

To improve the identification of genes differentially expressed in CNS HGNET-BCOR compared to the normal brain, we performed RNA transcriptome analysis (RNA-seq). This method is superior to microarrays because it has a larger dynamic range and it does not restrict the expression profile to known selected annotations. Indeed, RNA-seq has been shown to detect ~30% more differentially expressed genes than microarray analysis [15]. To take in consideration the tumor heterogeneity, whole-transcriptome sequencing was performed using fresh frozen tissues from two regions of the CNS HGNET-BCOR primary tumor and one inoculation metastasis. The development of inoculation metastases represents a mechanical displacement of the cells during the surgery rather than an active process and minor differences between the inoculation metastases and the primary tumor can be expected. Normal parietal brain was used as reference. The deregulated pathways are shown in Figure 5A. As previously described [1], among the activated pathways (orange), the basal cell carcinoma (BCC) pathway had the lowest p value. The BCC pathway is characterized by a cross-talk between the SHH and the WNT signaling according to the kyoto encyclopedia of genes and genomes (KEGG) [16]. According to the IPA, of the 72 molecules included in the BCC pathway, 13 were upregulated in the primary tumor sample no 127, 17 in the primary tumor sample no 123 and 14 in the inoculation metastasis no 166 (Figure 5B and Supplementary Table S1). The transcripts of the BCC pathway upregulated in our samples were as described by Sturm et al. and included target genes of the SHH pathway like *PTCH1* and *GLI1*, members of the *BMP*, *WNT* ligands and *WNT* ligand receptors families and of the *LEF1* and *TCF7L1* beta-catenin transcription complex. In addition, we found and validated (Supplementary Figure S1) an upregulation of *SMO*, which was not described before. Other pathways activated in both primary tumors included the glioblastoma multiforme signaling, the WNT/beta-catenin signaling and the PCP pathway. The PCP pathway was also activated in the metastasis. The glioblastoma multiforme signaling as described in the IPA, is characterized by the overexpression/amplification of Receptor Tyrosine Kinases (RTKs) and by the activation of the WNT signaling (Supplementary Table S2). In particular, we observed a strong expression of *IGF2* which is known to be required for SHH signaling [17]. The high expression of *IGF2* was maintained in the metastasis; moreover the expression of its receptor *IGF1R* was slightly upregulated in the primary tumor and the metastasis (Supplementary Table S3). The PCP pathway belongs to the non-canonical WNT pathways (Supplementary Table S4). In the metastasis, activity of mitotic polo-like kinases was predominant (Supplementary Table S5). Overexpression of members of the polo-like kinases family was observed also in the primary tumor, but at a lesser extent compared to the metastasis (Supplementary Table S6).

### The basal cell carcinoma pathway is homogenously activated in CNS HGNET-BCOR

**Figure 6:**
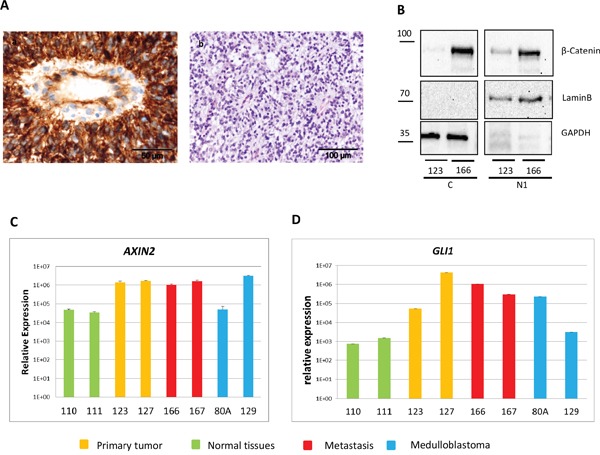
The BCC pathway is activated in CNS HGNET-BCOR **A.** FFPE tissues were stained with a beta-catenin antibody (a) or HE (b). **B.** Nuclei fractions containing soluble proteins (N1) and cytosoplasma (C) isolated from the primary tumor (123) and a metastasis (166) were analyzed by western blot with a beta-catenin specific antibody or antibodies against different cellular compartments (Lamin B for nuclei, GAPDH for cytoplasma). **C-D.** Expression of *AXIN2* (C), and *GLI1* (D) was analyzed by qRT-PCR in 2 regions of the primary tumor (123,127, in yellow), two metastases (166, 167 in red), two medulloblastoma (80A, SHH subtype, 129, WNT subtype, in blue), and two normal brain regions (110, 111, in green). After normalization to the housekeeping gene *HPRT1*, the relative quantification value was expressed as 2^−ΔΔCt^. Expression analysis was done in triplicates.

Our results suggested a homogeneous activation of the BCC pathway. Moreover, previous data identified the BCC pathway as a common feature of CNS HGNET-BCOR [1]. Hence, we focused further on the validation of this pathway. The activation of the WNT pathway is associated with the translocation of beta-catenin to the nucleus, however not all CNS HGNET-BCOR specimens showed nuclear beta-catenin staining [1]. Using immunohistochemistry, we detected a beta-catenin nuclear localization in a significant fraction of the tumor cells in the primary tumor and a metastasis (Figure 6A and Supplementary Figure S2). The staining in the metastasis was more intense; however the fraction of nuclei showing accumulation seemed not to differ. The nuclear accumulation was confirmed by a western blot analysis (Figure 6B). Again, higher level of beta-catenin was detected in the inoculation metastasis compared to the primary tumor. The nuclear localization of beta-catenin is not sufficient to predict activation of the WNT pathway [18, 19]. As a surrogate marker for beta-catenin transcription activity, we analyzed the expression of *AXIN2,* a main target of the beta-catenin/TCF/LEF complex [20]. Upregulation of *AXIN2* in ITD-positive samples compared to the normal brain and a medulloblastoma of the SHH subtype was confirmed by qRT-PCR and was similar to the expression level found in a medulloblastoma of the WNT subtype (Figure 6C). The level of *AXIN2* was similar in the primary tumor and metastasis, suggesting a similar level of activation despite the difference in beta-catenin amount. To strengthen this assumption, we analyzed the expression of a further LEF responsive gene, *LEF1* [21], in the primary tumor and the metastases. Again, no difference in the expression level between the primary tumor and the metastases was observed (Supplementary Figure S3). To functionally validate the activation of the SHH pathway, we confirmed the upregulation of the transcription factor *GLI1*, a main target gene of the pathway, in ITD-positive samples compared to the normal brain and a medulloblastoma of the WNT subtype by qRT-PCR (Figure 6D). A medulloblastoma sample of the SHH subtype also expressed high level of *GLI1*.

### Mutation analysis of key components of the SHH pathway reveals a Single Nucleotide Polymorphism (SNP) in *PTCH1*

**Figure 7:**
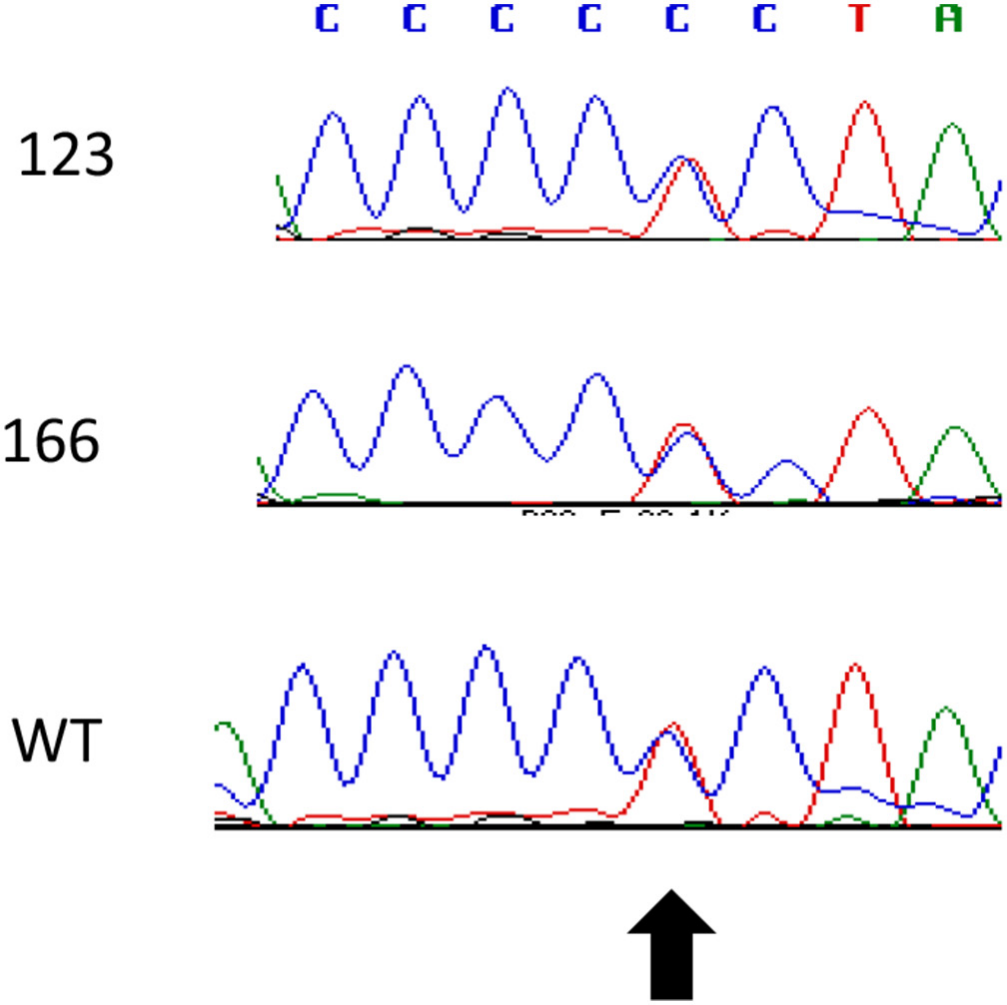
Detection of a *PTCH1* polymorphism The sequence of exon 23 of *PTCH1* was analyzed by Sanger sequencing in the genomic DNA from blood (WT), primary tumor (123) and metastasis (166). The arrow indicates the rs357564.

The mutation-driven activation of the SHH pathway has been described in basal cell carcinoma and medulloblastoma [22]. We analyzed the coding region of *SMO* by Sanger sequencing and of *PTCH1* and *SUFU* using a targeted sequencing approach. We identified a heterozygous mutation in *PTCH1* (NM_000264.3:c.3944C>T) in the genomic DNA extracted from the blood, from the primary tumor and from a metastasis (Figure 7). This non-synonymous SNP leads to a substitution of leucine for proline at the position 1315 in the C-terminal domain of *PTCH1* (NP_000255.2:p.Pro1315Leu) and it is predicted to be probably damaging according to Polyphen (score 0.915). This variant corresponds to the SNP rs357564 and has a MAF of 0.39. No missense mutations in *SUFU* and *SMO* were detected.

### Arsenic trioxide reduces the viability of a short-term cell culture of CNS HGNET-BCOR

**Figure 8:**
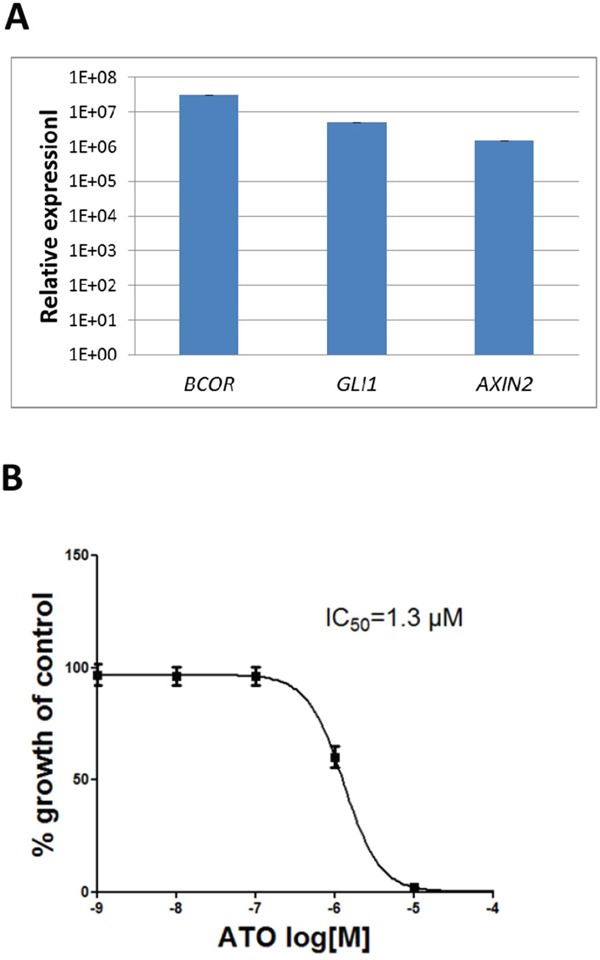
ATO reduces the viability of a short-term CNS HGNET-BCOR cell culture **A.** Expression of *BCOR*, *GLI1* and *AXIN2* in the PhKh1 cell culture at passage 4 was analyzed by qRT-PCR. After normalization to the housekeeping gene *HPRT1*, the relative quantification value was expressed as 2^−ΔΔCt^. **B.** PhKh1 cells were treated with ATO at doses from 1 nM to 100 μM. The logarithm of the Molarity (log [M]) is displayed on the X axis. The % of viable cells compared to the control treated with vehicle alone is shown on the Y axis. The data were fit to a sigmoidal dose-response curve using the GraphPad software. A representative experiment of three independent experiments is shown.

We established a primary culture from a metastasis of the CNS HGNET-BCOR patient (PhKh1). At passage 4, the same ITD found in the primary tumor and the metastasis was detectable, while the *BCOR* wild type allele was not detectable (data not shown). *BCOR*, *GLI1* and *AXIN2* were upregulated at a level similar to that found in the metastasis (Figure 8A) suggesting that high *BCOR* expression and the activation of the BCC pathway is maintained in this short-term cell culture. Arsenic trioxide is known to inhibit the SHH pathway at the level of GLI proteins [23]. We incubated the PhKh1 cells with different ATO concentrations (Figure 8B). ATO was able to reduce the cell viability with an IC_50_ of 1.3 μM.

## DISCUSSION

Our work functionally validated a homogenous activation of the basal cell carcinoma pathway in CNS-HGNET BCOR and delivered a rational for implementing ATO in the treatment protocols of these patients.

Tumors can display high levels of molecular heterogeneity [24]. This heterogeneity allows cancer cells to adapt to any hostile environment reducing the efficacy of targeted therapies that focuses only on one pathway. Our work showed that the BCC pathway is active in specimens isolated from different regions of the primary tumor and in different inoculation metastases supporting the relevance of targeted therapies affecting this pathway.

GLI transcription factors are the terminal effectors of the SHH pathway and lead to the upregulation of many genes associated with cancer growth and progression. Arsenic trioxide has been shown to reduce GLI activity by direct binding to GLI proteins reducing the viability of cell lines dependent on GLI, such as Ewing sarcoma and medulloblastoma [23]. Accordingly, ATO was able to affect the viability of a short-term CNS-HGNET BCOR cell culture. This result is particularly relevant because ATO is a FDA-approved drug for the treatment of acute promyelocytic leukemia (APL) and an extensive experience already exists in its application also to pediatric patients [25, 26]. In APL patients treated with ATO, total plasma arsenic concentrations can range from 862 to 3236 nM [27] with an acceptable safety profile. ATO penetrates the CNS of patients with APL with a mean concentration of 199 nM [27]. This concentration can be increased to levels comparable to those in blood by the addition of mannitol [28]. Importantly, 1.0 μM ATO showed little influence on the viability of cortical neurons [29]. Thus, therapeutically meaningful and safe concentrations of arsenic in plasma and CNS can be achieved. Moreover, ATO sensitizes glioblastoma cells to radiotherapy and chemotherapy [30, 31] suggesting that combination therapies including ATO may increase the efficacy of standard treatments.

We detected an upregulation of *SMO* that was not previously described by Sturm et al. However, in that work only recurrent alterations were shown and *SMO* upregulation may be patient-specific. *SMO* alterations leading to the activation of the SHH pathway in medulloblastoma are generally related to activating mutations [32]. Overexpression of *SMO* has been described in glioma where it associates with a worse survival [33]. SMO has been the primary target for the development of SHH-pathway inhibitors because SMO inhibition prevents the downstream activation of GLI transcription factors. Thus Vismodegib (GDC-0449) [34] and Erismodegib (NVP-LDE225) [35], both FDA approved drugs for the treatment of the basal cell carcinoma, could became relevant in the treatment of CNS-HGNET-BCOR, possibly in combination with ATO.

The reasons for the activation of the BCC pathway in CNS HGNET-BCOR are not understood so far. Activating mutations in the key components of the SHH pathway *PTCH1*, *SMO* and *SUFU* have been described in medulloblastoma [32] and basal cell carcinoma [36, 37]. In our patient, we detected a heterozygous mutation in *PTCH1* (rs357564). This variant was not described by Sturm et al [1]. However, in that work only recurrent molecular alterations detected in the four new CNS tumor entities were reported. This non-synonymous SNP affect the C-terminal domain of PTCH1 which is required for the inhibition of SMO [38]. This polymorphism was already reported in cancers of the skin [39], and in odontogenic keratocyst [40]. The biallelic variant c.3944T of the *PTCH1* gene is significantly associated with breast carcinoma [41]. Notably, the *PTCH1* variant c.3944C (Pro/Pro) may confer an increased risk for BCC in the population and an increased individual risk for multiple BCC [42]. Further work is needed to clarify the frequency and the role of this variant in the biology of CNS-HGNET-BCOR.

The SHH pathway plays a crucial role in the normal development of the cerebellum, in particular in granule neuron precursors (GNP) [43]. BCOR suppresses the tumor progression of SHH medulloblastoma by a BCL6/BCOR/SIRT1 complex that induces epigenetic repression of *GLI1* and *GLI2* [44]. BCOR forms a complex including polycomb group ring finger 1 (PCGF1) and is recruited to non-methylated CpG islands, where it induces repressive histone H2A monoubiquitylation [11]. The motive for binding to PCGF1, called PCGF Ub-like fold discriminator (PUFD), is localized between aa1634 and aa 1748 [6] and is therefore affected by the ITD. It is intriguingly to speculate that the ITDs disrupt the structure of the complex and leads to a derepression of the *GLI1* and *GLI2* promoters, underlining the relevance of GLI inhibitors such as ATO for the treatment of HGNET-BCOR patients. In the present work we showed that BCOR is expressed also at the protein level in CNS HGNET-BCOR, supporting the assumption of a functional significance of this protein in the biology of this tumor entity. While the present article was under review, a paper was published, which suggests that BCOR ITDs are likely to alter PCGF1 binding affinity [45].

In conclusion, our comprehensive analysis provides new information for further, individualized, innovative treatment options for this new entity of brain tumor. This is of great interest especially since it is a rare and highly malignant brain tumor with no existing standard therapy.

## MATERIALS AND METHODS

### Tissue samples and cell lines

Blood and tumor samples from the CNS HGNET-BCOR patient and an informed consent from the patient's parents were obtained before the genetic analysis. Two regions from the primary tumor (no 123 and no 127) and samples from two metastases (no 166 and no 167) were obtained during standard surgery. Formal approval of the local ethics committee for this study was not required, as this was a single case investigation. Tumor samples of two medulloblastoma patients (no 80a: SHH subtype, no 129: WNT subtype) were obtained as surplus material during standard surgery. This study was performed in agreement with the declaration of Helsinki on the use of human material for research. In accordance with the ethics committee of Rhineland-Palatinate, patient's parents agreed with the scientific use of the surplus material and no further approval of the medical ethics committee was required as the data were analyzed anonymously. RNA of normal brain tissues (adult frontal lobe no 110 and adult parietal lobe no 111) was sourced from commercial vendors (Biocat, Heidelberg, Germany). HEK-293 cells were cultured in Dulbecco's modified Eagle's medium (DMEM) containing 10% fetal calf serum. For the isolation of a primary cell culture (PhKh1) the fresh tumor sample derived from a metastasis was mechanically reduced to small pieces with the GentleMACS Dissociator (Miltenyi Biotec GmbH, Bergisch-Gladbach, Germany) and the cells were dissociated with 0.25% Trypsin. The cells were cultured in DMEM medium containing 10% Human Serum, 1% L-Glutamine and 1% Penicillin-Streptomycin (all Sigma-Aldrich, Taufkirchen, Germany). Cells at passage 4 were used for further experiments.

### Histology and immunohistochemistry

Hematoxylin and eosin (HE) staining was done according to standard protocols. Staining of beta-catenin was as previously described [46].

### Nucleic acid extraction

Tumor samples were analyzed by a pathologist and regions containing vital tumor cells were isolated for further processing. DNA from blood and tissues was extracted using Gentra Puregene Blood Kit (Qiagen, Hilden, Germany) following the manufacturer's instructions. RNA isolation was conducted using the RNeasy Lipid Tissue Mini Kit (Qiagen) and the Tissue Lyser (Qiagen) to disrupt the tumor. RNA was converted to cDNA by using PrimeScript RT Reagent Kit with gDNA Eraser (Takara Bio Europe, Saint-Germain-en-Laye, France). Quality control was performed using a Bioanalyser2100 (Agilent Technologies, Waldbronn, Germany). Only RNAs with a RIN value > 7 were used for RNA sequencing analysis and qRT-PCR.

### DNA sequencing

The coding exons of *BCOR* and *SMO* were amplified by PCR using 50 ng of DNA from blood and tumor (see Supplementary Table S7 and S8 for primers). The cDNA region containing the *BCOR* ITD was amplified using primers 5′-GGCAGCTCTGTTTGTGAACC and 5′-TCTAAGTCCTTCTGACTGGA. PCR reactions were performed using 0.1U of Taq Polymerase (Axon Labortechnik, Kaiserlautern, Germany). 0.4 mM of each primer, 200 mM dNTP mix, 1.5 mM MgCl2 as well as 0.2 M betaine and the following PCR conditions: an initial denaturation step at 94°C for 5 min, 36 cycles at 94°C for 30 s, 60°C for 30 s, 72°C for 60 s, and a final extension step at 72°C for 10 min. PCR products were purified by an enzymatic method using 10 U exonuclease I and 2 U shrimp alkaline phosphatase (New England Biolabs, Frankfurt, Germany) for 30 min at 37°C and 15 min at 80°C and sequenced by using ABI Prism 3100 Genetic Analyser and the BigDye v3 Terminator Kit (Thermo Fisher, Dreieich, Germany). The sequences were compared to the reference sequence using the Sequencher program (Gene Codes, USA). Mutation analysis of *SUFU* and *PTCH1* was performed by Next Generation Sequencing using the TruSight Cancer panel (Illumina, San Diego, FC-121-0202). 50 ng DNA was used for the library construction according to the manufacturer's instructions (Nextera Rapid Capture). Paired-end sequencing was performed on an Illumina NextSeq 500 platform (2x150 cycles). Data were processed using BWA Enrichment v1.0 for the generation of BAM files and GATK for variant calling. Analysis of variants was performed with the VariantStudio software (Illumina). For Sanger sequencing validation, primers were designed encompassing each identified alteration.

### qRT-PCR

qRT-PCR was performed using the Light Cycler 480 II detection system and software (Applied Biosystems, Darmstadt, Germany) with KAPA SYBR FAST Light Cycler 480 Kit (PeqLab, Erlangen, Germany). Following primers were used: *BCOR*: 5′- GGCAGCTCTGTTTGTGAACC and 5′- CCTGAGCCACAGATACTTGG; *GLI1*: 5′- AGCTTG TCCCACACCGGTAC and 5′- GAGGATGCTCCAT TCTCTGGTG; *AXIN2*: 5′-GCTCAGAGCTTGACCCTGG and TCATACATCGGGAGCACCGT; *LEF1*: 5′-GAAGC CTCAGCATGAACAGAGAAA and 5′- ATAATATTTA GCCTGCTCTTCACGG; *SMO*: 5′-CCCTGTGGCA ACTCCAGTG and 5′-CAGCCACCAGGCATTTCTGC; *HPRT1*: 5′-TGACACTGGCAAAACAATGCA and 5′-GGTCCTTTTCACCAGCAAGCT. After normalization to the housekeeping gene *HPRT1*, the relative quantification value was expressed as 2^−ΔΔCt^. The calibrator was calculated as the maximal number of cycles used in the PCR (40) minus the mean of the *HPRT1* Ct values, resulting in a value of 19.

### RNA sequencing

Libraries were constructed using the TruSeq mRNA stranded protocol (Illumina) using 2 μg total RNA. Pair end sequencing was performed on a NextSeq500 Instrument (Illumina). 50 million reads per library were produced. The reads were trimmed to a maximum read length of 125 bp. TruSeq adapter sequences were trimmed. Read mapping was performed using the TopHat2 v2.0.7 aligner [47] and the Homo sapiens UCSC hg19 reference genome (RefSeq gene annotations). FPKM values of reference genes and transcripts were estimated by Cufflinks 2.0.5 [48]. TPM values of genes and transcripts were calculated according to Lior Pachter (https://arxiv.org/abs/1104.3889)

### Pathway analysis

Ingenuity Pathway Analysis (IPA; Ingenuity Systems/Qiagen, Redwood City, USA) was used to map lists of significant genes to gene ontology groups and biological pathways. An Ingenuity ‘core analysis’ typically generates biological functions, canonical pathways and functional molecular networks pertaining to the dataset in question based on the Ingenuity Pathway Knowledge Base (IPKB) which is derived from known functions and interactions of genes published in the literature. To identify deregulated pathways, we first calculated the ratio of the expression between the TPM of the tumor tissues and of the normal parietal brain and selected the genes with a fold change >10. IPA then uses Fisher's exact test to calculate a probability value to indicate the association between each gene in the list and IPA-curated pathways and biological functions. Functions and pathways with *p*-value < 0.05 were considered to be statistically significant.

### Preparation and analysis of subcellular compartments

Nuclear and cytoplasmatic extracts were generated according to Holden and Horton [49]. Briefly, fresh frozen tissues were pulverized using the TissueLyser (Qiagen). The pulver was incubated for 30 minutes in 400 μl of buffer 2 (150 mM NaCl, 50 mM HEPES pH 7.4, 1% NP-40). After centrifugation at 7000 rcf for 10 minutes, the supernatant containing the cytoplasm was isolated and the pellet containing the nuclei was resuspended in 400 μl of buffer 3 (150 mM NaCl, 50 mM HEPES pH 7.4, 0.5 % sodium deoxycholate, 0.1 % sodium dodecyl sulfate) and incubated at 4°C for 1 hour. After centrifugation, the supernatant contained soluble nuclear extract and the pellet contained insoluble proteins of the nuclear fraction. The pellet was resuspended in 200 μl of buffer 4 (150 mM NaCl, 50 mM HEPES pH 7.4, 0.5% sodium deoxycholate, 1% sodium dodecyl sulfate, 100 mM dithiothreitol). HEK-293 cells were detached by trypsinization. After centrifugation, the protein extraction of the cell pellet was performed as described above with the pulverized fresh frozen tissue. Protein concentration of samples was measured by Bradford Assay. Equal amounts were loaded onto 10 % SDS-PAGE gels and electrotransferred onto nitrocellulose membrane (0.1 μm, GE Healthcare, Freiburg, Germany). Immunostaining was performed with primary antibody followed by detection with horseradish-peroxidase conjugated antibodies (Anti-mouse IgG, Cell Signaling, (Leiden, The Netherland), 7076S; Anti-rabbit IgG, KPL, Inc (Gaithersburg, USA), 71-00-30; Anti-goat IgG, Santa Cruz (Heidelberg, Germany), sc-2953. A polyclonal antibody against BCOR was purchased from abcam (Cambridge, UK) (ab88112). Lamin B and GAPDH were from Cell Signaling (9166 and 2118) and beta-Catenin from Sigma-Aldrich (Taufkirchen, Germany) (C7202). The blots were washed with PBS-T and blocked with 5 % skim milk. Chemiluminescence analysis was done using ECL Western Blotting Detection Reagent (GE Healthcare) or WesternBright Sirius (Biozym Scientific GmbH, Hessisch Oldendorf, Germany) in the ChemiLux Imager CsX-1400M system (Intas, Göttingen, Germany).

### Cellular proliferation assays

ATO (Sigma-Aldrich) was dissolved at a concentration of 5 mM in PBS using sodium hydroxide and dilute hydrochloric acid to adjust to pH 7.6. Cells were plated in triplicates at a density of 5.000 cells/well in a 96-well plate. ATO at varying concentrations or vehicle alone were added to the cells. Viable cells were quantified using the cell proliferation reagent WST-1 (Roche, Mannheim, Germany) according to the manufacturer's protocol after 3 days. Dose-response curves were plotted to determine the half-maximal inhibitory concentration (IC_50_) using the GraphPad Prism v.5 (GraphPad Software, San Diego CA, USA).

## SUPPLEMENTARY TABLES AND FIGURES




