# Supplementary material for: Activation of the basal cell carcinoma pathway in a patient with CNS HGNET-BCOR diagnosis: consequences for personalized targeted therapy
Source: Oncotarget. 2016 Nov 4;7(50):83378–91. doi: 10.18632/oncotarget.13092 (PMC5347776; doi:10.18632/oncotarget.13092)
Supplement: Supplementary file 1 [file oncotarget-07-83378-s001.pdf]

Activation of the basal cell carcinoma pathway in a patient with CNS HGNET-BCOR diagnosis: consequences for personalized targeted therapy

SUPPLEMENTARY TABLES AND FIGURES

Supplementary Table S1: Transcripts of the BCC pathway upregulated in the primary tumor and a metastasis

See Supplementary File 1

Supplementary Table S2: Transcripts of the Glioblastoma Multiforme Pathway upregulated in sample 127. See supplementary Table 1 for legend

Symbol	Entrez Gene Name	Exp Fold Change	Expected	Location	Type(s)
CCND1	cyclin D1	14.102	Up	Nucleus	transcription regulator
CDK6	cyclin-dependent kinase 6	13.666		Nucleus	kinase
CDKN1A	cyclin-dependent kinase inhibitor 1A (p21. Cip1)	10.620	Down	Nucleus	kinase
EGFR	epidermal growth factor receptor	16.588	Up	Plasma Membrane	kinase
FZD7	frizzled class receptor 7	11.610	Up	Plasma Membrane	G-protein coupled receptor
FZD10	frizzled class receptor 10	36.247	Up	Plasma Membrane	G-protein coupled receptor
IGF2	insulin like growth factor 2	196.468	Up	Extracellular Space	growth factor
LEF1	lymphoid enhancer binding factor 1	28.303	Up	Nucleus	transcription regulator
PDGFRA	platelet derived growth factor receptor alpha	15.102	Up	Plasma Membrane	kinase
PLCE1	phospholipase C epsilon 1	10.518	Up	Cytoplasm	enzyme
PLCG2	phospholipase C gamma 2	24.915	Up	Cytoplasm	enzyme
SMO	smoothened. frizzled class receptor	13.632	Up	Plasma Membrane	G-protein coupled receptor
WNT11	wingless-type MMTV integration site family member 11	94.040	Up	Extracellular Space	other
WNT5A	wingless-type MMTV integration site family member 5A	16.129	Up	Extracellular Space	cytokine

Supplementary Table S3: Expression of IGF2 and IGF1R in the primary tumor, a metastasis and normal brain

Sample	Symbol	Entrez Gene Name	TPM
127	IGF2	insulin like growth factor 2	1139.32
127	IGF1R	insulin-like growth factor 1 receptor	72.77
123	IGF2	insulin like growth factor 2	9944.16
123	IGF1R	insulin-like growth factor 1 receptor	44.8
166	IGF2	insulin like growth factor 2	1100.83
166	IGF1R	insulin-like growth factor 1 receptor	35.52
111	IGF2	insulin like growth factor 2	5.79
111	IGF1R	insulin-like growth factor 1 receptor	11.11

123 and 127 are two regions of the primary tumor. 166 is a metastasis. 111 is normal brain. The TPM values of the indicated transcripts are shown.

Supplementary Table S4: Transcripts of the PCP Pathway upregulated in sample 127. See supplementary Table 1 for legend

Symbol	Entrez Gene Name	Exp Fold Change	Expected	Location	Type(s)
CELSR1	cadherin EGF LAG seven-pass G-type receptor 1	63.767		Plasma Membrane	G-protein coupled receptor
FZD7	frizzled class receptor 7	11.610	Up	Plasma Membrane	G-protein coupled receptor
FZD10	frizzled class receptor 10	36.247	Up	Plasma Membrane	G-protein coupled receptor
MAPK9	mitogen-activated protein kinase 9	14.183	Up	Cytoplasm	kinase
ROR2	receptor tyrosine kinase-like orphan receptor 2	15.585	Up	Plasma Membrane	kinase
SDC1	syndecan 1	12.272		Plasma Membrane	enzyme
SMO	smoothened. frizzled class receptor	13.632	Up	Plasma Membrane	G-protein coupled receptor
WNT11	wingless-type MMTV integration site family member 11	94.040	Up	Extracellular Space	other
WNT5A	wingless-type MMTV integration site family member 5A	16.129	Up	Extracellular Space	cytokine

Supplementary Table S5: Transcripts of the Polo like kinases Pathway upregulated in the metastasis (sample 166). See supplementary Table 1 for legend

Symbol	Entrez Gene Name	Exp Fold Change	Expected	Location	Type(s)
CCNB1	cyclin B1	10.047	Up	Cytoplasm	kinase
CCNB2	cyclin B2	88.049	Up	Cytoplasm	other
CDC20	cell division cycle 20	12.053	Up	Nucleus	other
CDC25A	cell division cycle 25A	19.930	Up	Nucleus	phosphatase
CDK1	cyclin-dependent kinase 1	160.964	Up	Nucleus	kinase
CHEK2	checkpoint kinase 2	12.647		Nucleus	kinase
ESPL1	extra spindle pole bodies like 1. separase	12.644	Down	Nucleus	peptidase
FBXO5	F-box protein 5	11.876	Down	Nucleus	enzyme
KIF11	kinesin family member 11	41.834	Up	Nucleus	other
KIF23	kinesin family member 23	41.785	Up	Cytoplasm	other
PLK1	polo-like kinase 1	20.185	Up	Nucleus	kinase
PLK4	polo-like kinase 4	23.206	Up	Cytoplasm	kinase
PPP2R3B	protein phosphatase 2 regulatory subunit B". beta	18.921		Nucleus	phosphatase
PRC1	protein regulator of cytokinesis 1	13.463		Nucleus	other
PTTG1	pituitary tumor-transforming 1	15.885	Down	Nucleus	transcription regulator
WEE1	WEE1 G2 checkpoint kinase	93.430	Down	Nucleus	kinase

Supplementary Table S6: Transcripts of the Polo like kinases Pathway upregulated in sample 127. See supplementary Table 1 for legend

Symbol	Entrez Gene Name	Exp Fold Change	Expected	Location	Type(s)
CCNB1	cyclin B1	1.324	Up	Cytoplasm	kinase
CCNB2	cyclin B2	9.24	Up	Cytoplasm	other
CDC20	cell division cycle 20	1.053	Up	Nucleus	other
CDC25A	cell division cycle 25A	1.258	Up	Nucleus	phosphatase
CDK1	cyclin-dependent kinase 1	20.525	Up	Nucleus	kinase
CHEK2	checkpoint kinase 2	4.239		Nucleus	kinase
ESPL1	extra spindle pole bodies like 1. separase	1.826	Down	Nucleus	peptidase
FBXO5	F-box protein 5	2.212	Down	Nucleus	enzyme
KIF11	kinesin family member 11	7.833	Up	Nucleus	other
KIF23	kinesin family member 23	4.541	Up	Cytoplasm	other
PLK1	polo-like kinase 1	5.515	Up	Nucleus	kinase
PLK4	polo-like kinase 4	3.235	Up	Cytoplasm	kinase
PPP2R3B	protein phosphatase 2 regulatory subunit B". beta	1,112		Nucleus	phosphatase
PRC1	protein regulator of cytokinesis 1	2.860		Nucleus	other
PTTG1	pituitary tumor-transforming 1	2.211	Down	Nucleus	transcription regulator
WEE1	WEE1 G2 checkpoint kinase	204.071	Down	Nucleus	kinase

Supplementary Table S7: Primers used for the Sanger Sequencing of BCOR

Primer name	Sequence 5‘-3‘
BCOR-E1-F	ATGCCTCAGTTTCTCACTCTG
BCOR-E1-R	TAGGAGAACTCAAAACAACTCG
BCOR-E2-F	AGCGCGGGAGCTGCTGAG
BCOR-E2-R	GCGCGAGGCAAAAGCTTCG
BCOR-E3-F	GACCACTGTAGACAGCAGG
BCOR-E3-R	ATCAAGAGCGGCCTACTAGG
BCOR-E4-F	GGGCCATTGACAGCTGGTG
BCOR-E4-R	CAGCCCTGCTCTGAACTGC
BCOR-E5-F1	TGTGCTTGGATGTTAAGGCTG
BCOR-E5-R1	GGACCGACGTAGTGAGGTG
BCOR-E5-F2	ATTCACTGAACATGTACAAGGC
BCOR-E5-R2	AGCCCTGCTGTGAACCAGG
BCOR-E5-F3	CTCCTCTGTTGGAGAAGCAG
BCOR-E5-R3	CTGGGTGAGGGTAGACAGG
BCOR-E5-F4	TCTCCAAATGAGGCATTCAGG
BCOR-E5-R4	AAGTTACAGCAGGGCCATCG
BCOR-E5-F5	CCCTGCAGCAGCACCGTG
BCOR-E5-R5	GCATCCAGACACGCCTCAG
BCOR-E6-F	GAGTATGTATTTAACTTGCTAGTG
BCOR-E6-R	CAATGAATAAAGACGACCATGG
BCOR-E7-F	AGTTACATCTCATAAGCTGTGG
BCOR-E7-R	CACAGACGCCTCGTTCCTG
BCOR-E8-F	TTGTGCTAACCATGTGTGAGG
BCOR-E8-R	CGAGGCAGTGTGGGAATGG
BCOR-E9-10-F	AGATGGCCAAAGATGATTAGG
BCOR-E9-10-R	CTGAGGCTGGAATGAAGGTG
BCOR-E11-F	GCCGCTCCAGTGATCAGTG
BCOR-E11-R	GACATGCTCCTCTAGGACAG
BCOR-E12-F	AATAAAGTGCCTCTCTCCACG
BCOR-E12-R	AGAGACAGCAGTGAGAGTGG
BCOR-E13-F	TCAGGCGCTTCGCACATGTG
BCOR-E13-R	TCAGAAGCTTTGGTTCACAGG
BCOR-E14-F	AGTTTCCCTATCTGTGCAGTG
BCOR-E14-R	CTTTACAGTTTCAGCCTTTGTG
BCOR-E15-F	GGAAATGACCTTCCAGCCTG
BCOR-E15-R	ACTACAGCAAGATGTCCACAG
BCOR-E16-F	TTTCTGTCTGGTATGGGTGAG
BCOR-E16-R	AAATATCTACTGCATTGTGTGAG
BCOR-E17-F	GATCAGAGAGTAGGTATGGTG
BCOR-E17-R	TCTAAGTCCTTCTGACTGGAG

Supplementary Table S8: Primers used for the Sanger sequencing of SMO

Exon1	GAGGTCGTGCGTGTGGC
	GCCTGAGCCTGTTCTTTCC
Exon2	GGAGGACAGGGGTGAAGC
	CCTGCCCTATACCCGGTC
Exon3	TTCCCTAAACAAGAGGCTCG
	CTTCCCTCTGGCTCTCTTCC
Exon4	GGTCTGGGGCTCAGTTAAGG
	GGTAAACGCACACTTCAGCC
Exon5-6	GGGAGACCAGGTAGAGGGAG
	CTGGGGTTTCAGCATGGG
Exon7	ACTCACCCATCCTTCCCAG
	AGGAGGGTGCAGTGTTGG
Exon 8	GAGGTGGGTGTCTTTATGGC
	GACTCTCTCCTCCCCACTGC
Exon 9	GGTGACAGAGCAAGATCCTATCTC
	GTCCCAAGGCTGAGGGTG
Exon10	TCCCAAGATTTGATGGGAAG
	CCAAATAATCTGTGTGCCCC
Exon11	CAGGGACCGGGAAGTCAC
	ACAGCCCCTGTACTCCAGTC
Exon12	ACAGGTTAAGTGCTCCCAGG
	GAAGAAGAGCCTTGAAGGTATTGG

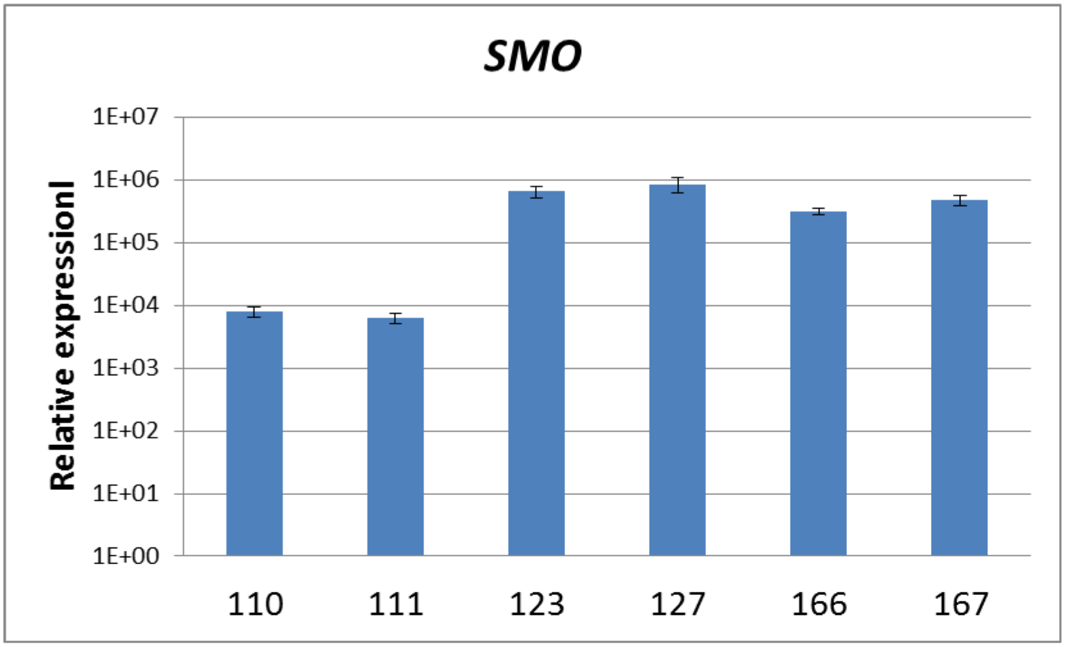

Supplementary Figure S1: The expression of *SMO* was analyzed by qRT-PCR in two normal brain tissues (110 and 111), in two regions of the primary tumor (123 and 127) and in two inoculation metastases (166 and 167). After normalization to the housekeeping gene *HPRT1*, the relative quantification value was expressed as $2^{-\Delta\Delta Ct}$. Expression analysis was done in triplicates.

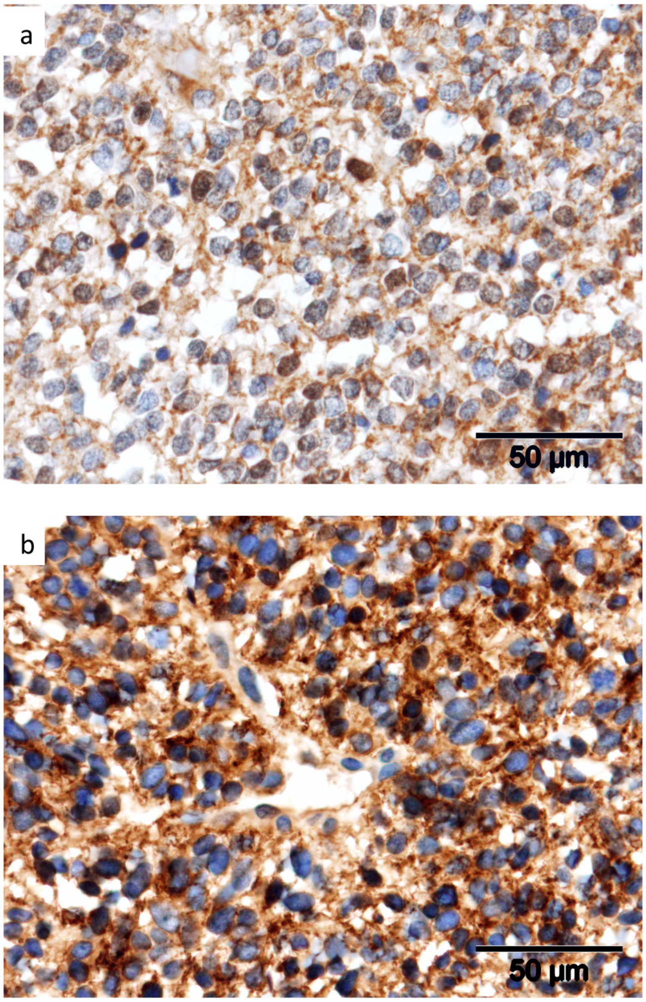

Supplementary Figure S2: The primary tumor a. and a metastasis b. were stained in parallel with a beta-catenin specific antibody.

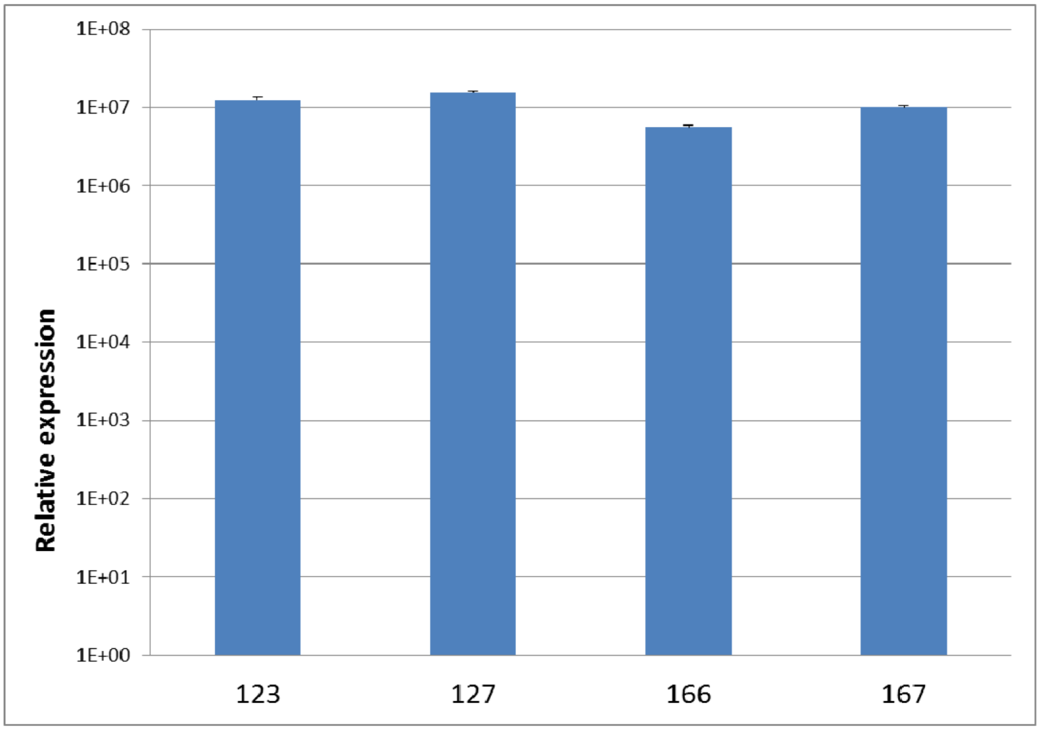

Supplementary Figure S3: The expression of LEF1 was analyzed by qRT-PCR in two regions of the primary tumor (123, 127) and in two inoculation metastases (166 and 167). After normalization to the housekeeping gene *HPRT1*, the relative quantification value was expressed as $2^{-\Delta\Delta Ct}$. Expression analysis was done in triplicates.
