# Supplementary material for: Activation of the basal cell carcinoma pathway in a patient with CNS HGNET-BCOR diagnosis: consequences for personalized targeted therapy
Source: Oncotarget. 2016 Nov 4;7(50):83378–91. doi: 10.18632/oncotarget.13092 (PMC5347776; doi:10.18632/oncotarget.13092)
Supplement: Supplementary file 2 [file oncotarget-07-83378-s002.doc]

**Supplementary Table 1**. Transcripts of the BCC pathway upregulated in the primary tumor and a metastasis

| **Sample** | **Symbol** | **Entrez Gene Name** | **Exp Fold Change** | **Expected** | **Location** | **Type(s)** |
| --- | --- | --- | --- | --- | --- | --- |
| 127 | BMP1 | bone morphogenetic protein 1 | 11.426 | Up | Extracellular Space | peptidase |
| 127 | BMP6 | bone morphogenetic protein 6 | 17.645 | Up | Extracellular Space | growth factor |
| 127 | FZD7 | frizzled class receptor 7 | 11.610 | Up | Plasma Membrane | G-protein coupled receptor |
| 127 | FZD10 | frizzled class receptor 10 | 36.247 | Up | Plasma Membrane | G-protein coupled receptor |
| 127 | GLI1 | GLI family zinc finger 1 | 20.756 | Up | Nucleus | transcription regulator |
| 127 | GLI2 | GLI family zinc finger 2 | 39.318 | Up | Nucleus | transcription regulator |
| 127 | LEF1 | lymphoid enhancer binding factor 1 | 28.303 | Up | Nucleus | transcription regulator |
| 127 | PTCH1 | patched 1 | 20.700 |  | Plasma Membrane | transmembrane receptor |
| 127 | SMO | smoothened. frizzled class receptor | 13.632 | Up | Plasma Membrane | G-protein coupled receptor |
| 127 | STK36 | serine/threonine kinase 36 | 15.644 | Up | Cytoplasm | kinase |
| 127 | TCF7L1 | transcription factor 7-like 1 (T-cell specific. HMG-box) | 28.964 | Up | Nucleus | transcription regulator |
| 127 | WNT11 | wingless-type MMTV integration site family member 11 | 94.040 | Up | Extracellular Space | other |
| 127 | WNT5A | wingless-type MMTV integration site family member 5A | 16.129 | Up | Extracellular Space | cytokine |
| 123 | BMP4 | bone morphogenetic protein 4 | 15.219 | Up | Extracellular Space | growth factor |
| 123 | FZD2 | frizzled class receptor 2 | 16.375 | Up | Plasma Membrane | G-protein coupled receptor |
| 123 | FZD7 | frizzled class receptor 7 | 12.853 | Up | Plasma Membrane | G-protein coupled receptor |
| 123 | FZD10 | frizzled class receptor 10 | 32.079 | Up | Plasma Membrane | G-protein coupled receptor |
| 123 | GLI1 | GLI family zinc finger 1 | 25.586 | Up | Nucleus | transcription regulator |
| 123 | GLI2 | GLI family zinc finger 2 | 51.690 | Up | Nucleus | transcription regulator |
| 123 | LEF1 | lymphoid enhancer binding factor 1 | 39.641 | Up | Nucleus | transcription regulator |
| 123 | PTCH1 | patched 1 | 17.972 |  | Plasma Membrane | transmembrane receptor |
| 123 | SMO | smoothened. frizzled class receptor | 13.414 | Up | Plasma Membrane | G-protein coupled receptor |
| 123 | STK36 | serine/threonine kinase 36 | 12.099 | Up | Cytoplasm | kinase |
| 123 | TCF3 | transcription factor 3 | 13.109 | Up | Nucleus | transcription regulator |
| 123 | TCF7L1 | transcription factor 7-like 1 (T-cell specific. HMG-box) | 90.952 | Up | Nucleus | transcription regulator |
| 123 | WNT4 | wingless-type MMTV integration site family member 4 | 11.293 | Up | Extracellular Space | cytokine |
| 123 | WNT6 | wingless-type MMTV integration site family member 6 | 17.261 | Up | Extracellular Space | other |
| 123 | WNT11 | wingless-type MMTV integration site family member 11 | 130.835 | Up | Extracellular Space | other |
| 123 | WNT5A | wingless-type MMTV integration site family member 5A | 12.258 | Up | Extracellular Space | cytokine |
| 123 | WNT5B | wingless-type MMTV integration site family member 5B | 12.092 | Up | Extracellular Space | other |
| 166 | BMP3 | bone morphogenetic protein 3 | 14.367 | Up | Extracellular Space | growth factor |
| 166 | BMP4 | bone morphogenetic protein 4 | 15.613 | Up | Extracellular Space | growth factor |
| 166 | BMP6 | bone morphogenetic protein 6 | 22.925 | Up | Extracellular Space | growth factor |
| 166 | FZD1 | frizzled class receptor 1 | 14.778 | Up | Plasma Membrane | G-protein coupled receptor |
| 166 | FZD7 | frizzled class receptor 7 | 20.290 | Up | Plasma Membrane | G-protein coupled receptor |
| 166 | FZD10 | frizzled class receptor 10 | 70.625 | Up | Plasma Membrane | G-protein coupled receptor |
| 166 | GLI1 | GLI family zinc finger 1 | 63.334 | Up | Nucleus | transcription regulator |
| 166 | GLI2 | GLI family zinc finger 2 | 22.610 | Up | Nucleus | transcription regulator |
| 166 | LEF1 | lymphoid enhancer binding factor 1 | 29.348 | Up | Nucleus | transcription regulator |
| 166 | PTCH1 | patched 1 | 13.202 |  | Plasma Membrane | transmembrane receptor |
| 166 | SMO | smoothened. frizzled class receptor | 20.629 | Up | Plasma Membrane | G-protein coupled receptor |
| 166 | TCF7L1 | transcription factor 7-like 1 (T-cell specific. HMG-box) | 22.409 | Up | Nucleus | transcription regulator |
| 166 | WNT11 | wingless-type MMTV integration site family member 11 | 132.445 | Up | Extracellular Space | other |
| 166 | WNT5A | wingless-type MMTV integration site family member 5A | 18.931 | Up | Extracellular Space | cytokine |

123 and 127 are two regions of the primary tumor. 166 is a metastasis. The Exp fold change indicates the ratio between the TPMs of the tumor tissue and of the normal brain (sample 111). The expected status (upregulated or downregulated) if the pathway is activate is indicated. The location and function (Type) of the corresponding proteins are also indicated
